# A comparative analysis of intravenous infusion methods for low-resource environments

**DOI:** 10.3389/fmed.2024.1326144

**Published:** 2024-02-20

**Authors:** Oluwakemi Tomobi, Samantha Avoian, Ifeoma Ekwere, Shivani Waghmare, Fatima Diaban, Gabrielle Davis, Yacine Sy, Oluchi Ogbonna, Kevin Streete, Ebenezer Aryee, Vasanthini Kulasingham, John B. Sampson

**Affiliations:** ^1^Department of Anesthesiology, West Virginia University, Morgantown, WV, United States; ^2^Division of Health, Science, and Technology, Howard Community College, Columbia, MD, United States; ^3^University of Texas at Southwestern, Dallas, TX, United States; ^4^Department of Anesthesiology & Critical Care Medicine, Johns Hopkins School of Medicine, Baltimore, MD, United States; ^5^Howard University College of Medicine, Washington, DC, United States; ^6^Advocate Aurora Health, Downers Grove, IL, United States; ^7^Johns Hopkins University School of Medicine, Baltimore, MD, United States; ^8^University of Maryland School of Medicine, Baltimore, MD, United States

**Keywords:** cost-effectiveness analysis, global health disparities, critically ill, low-resource setting, intravenous infusion therapy, sedation

## Abstract

**Introduction:**

Intravenous (IV) therapy is a crucial aspect of care for the critically ill patient. Barriers to IV infusion pumps in low-resource settings include high costs, lack of access to electricity, and insufficient technical support. Inaccuracy of traditional drop-counting practices places patients at risk. By conducting a comparative assessment of IV infusion methods, we analyzed the efficacy of different devices and identified one that most effectively bridges the gap between accuracy, cost, and electricity reliance in low-resource environments.

**Methods:**

In this prospective mixed methods study, nurses, residents, and medical students used drop counting, a manual flow regulator, an infusion pump, a DripAssist, and a DripAssist with manual flow regulator to collect normal saline at goal rates of 240, 120, and 60 mL/h. Participants’ station setup time was recorded, and the amount of fluid collected in 10 min was recorded (in milliliters). Participants then filled out a post-trial survey to rate each method (on a scale of 1 to 5) in terms of understandability, time consumption, and operability. Cost-effectiveness for use in low-resource settings was also evaluated.

**Results:**

The manual flow regulator had the fastest setup time, was the most cost effective, and was rated as the least time consuming to use and the easiest to understand and operate. In contrast, the combination of the DripAssist and manual flow regulator was the most time consuming to use and the hardest to understand and operate.

**Conclusion:**

The manual flow regulator alone was the least time consuming and easiest to operate. The DripAssist/Manual flow regulator combination increases accuracy, but this combination was the most difficult to operate. In addition, the manual flow regulator was the most cost-effective. Healthcare providers can adapt these devices to their practice environments and improve the safety of rate-sensitive IV medications without significant strain on electricity, time, or personnel resources.

## 1 Introduction

Intravenous (IV) therapy is used to achieve homeostatic balance and is crucial to the care of critically ill patients. Intravenous fluids are required to maintain or increase cardiac output vital for tissue perfusion and for the sedation necessary to promote healing during admission to an intensive care unit (ICU) (1). Yet, excessive administration or under-delivery of fluids or medications can result in complications that contribute to morbidity and mortality (2). Furthermore, accuracy, cost, and the perspectives of healthcare workers with respect to the range of IV therapies available are still minimally explored in the low-resource setting.

In critically ill patients with hemodynamic instability, such as with distributive shock, appropriate fluid balance is crucial. Aggressive fluid administration can result in volume overload and worsen the patient’s condition (3–6). Similarly, sedation overdosage and underdosage can negatively affect health measures such as the depth of sedation for ventilator use or successful pain management to reduce hospital stay. Accurate infusion methods can improve recovery outcomes in the ICU and reduce hospital stay (7). Although different IV delivery methods may have different levels of accuracy, additional factors must be considered for low-resource environments.

Accuracy in delivering IV fluids and other medications is achieved by using infusion pumps and other volumetric devices. Many of these devices are expensive and require electricity. Most available infusion pumps range in price from $1,200 to $4,000 each (8) and may be prohibitively expensive in low-resource settings. To overcome these financial barriers, other devices may be considered.

Health providers in low-resource settings usually resort to drop-counting, which relies on raising the IV bag above a patient to increase the hydrostatic pressure to overcome the vein’s peripheral pressure, and observing the flow rate by counting the number of drops per minute (9). Inaccuracy of traditional drop-counting practices places patients at risk for complications that may increase morbidity and mortality (9).

The manual flow regulator is a circular device that allows the healthcare provider to manually set the flow rate in milliliters per hour (10). This device is advantageous because it facilitates a consistent infusion rate, does not require electricity or batteries, and costs $4.00 U.S. dollars (USD). A disadvantage, however, is that the accuracy may not approach that of the IV infusion pump.

A portable device known as the DripAssist has been shown in 2 studies to be accurate and can be used in low-resource settings such as prehospital and military medicine (11, 12). The DripAssist was developed to administer IV fluid infusions in low-resource areas at a low cost with no electricity requirements (11, 12). Each DripAssist device is priced at $400 USD and requires one AA battery. Though the DripAssist provides a low-cost option for achieving accuracy in low-resource settings, it is very sensitive to any movement.

According to the current literature, accuracy, precision, and setup time are crucial considerations when comparing medical devices (5). In low-resource environments, such as India and Senegal, both overall costs and running considerations, such as electricity reliance, should be considered (13–17). The perceptions of healthcare workers are additionally important when it comes to the use of devices (18, 19). In this prospective mixed methods comparative study, we aimed to compare the different methods available for IV infusions that require close volume and rate control. To this end, we (1) compared the accuracy, precision, and setup time over 3 drip goals of 5 IV infusion methods, (2) evaluated healthcare worker perception of these options, and finally (3) conducted a cost-effectiveness analysis of the devices. The ultimate goal of this analysis is to identify a device that bridges the gap between accuracy, cost, and electricity reliance for IV infusion methods in low-resource settings.

## 2 Methods

This study was a mixed-methods prospective trial and a cost-effectiveness analysis to compare IV infusion devices in terms of their use in 2 countries, Senegal and India. It was conducted at the Johns Hopkins University School of Medicine and Howard Community College and was approved by the Institutional Review Boards at both institutions (IRB00254064 and HCC − 2021-08-25, respectively). This approval included special permission to conduct research with institutional employees and special permission from the nursing department leadership to recruit nurses. Participants were informed about the study through a detailed consent form, which was signed prior to participation.

### 2.1 Participant recruitment

The study included a set of local health nurse, resident, and medical student volunteers. Inclusion criteria for participants included age ≥ 18 years, training or practicing with a minimum of a registered nurse (RN) license for nurses, a medical degree for resident trainees in the workforce, or enrollment in a U.S. medical school for medical students. Sample size determination was based on sufficient numbers needed for usability studies (at least 30 for quantitative analysis) (20). No patients were involved in the study. For this study, recruitment was targeted at local nurses who could participate at either the Johns Hopkin Hospital site or the Howard Community College site (both in Maryland) because nurses are typically responsible for administering the proper IV infusion rate prescribed by the doctor. Medical students and residents were also recruited to the study because residents are also part of the workforce in low-resource settings and because we wanted to investigate whether medical students with no experience could easily learn the IV infusion setup.

Exclusion criteria included nurses with less than an RN level of education, and undergraduate (pre-health) students.

Electronic flyers were created to advertise the study and to recruit participants. Participants were scheduled in advance or recruited onsite on the day of participation. Prior to the experiment, participants had the option of watching a demonstration of each IV infusion method through a series of videos made by the researchers. They also had the option of observing a 5-min, live, in-person didactic and demonstration session of each IV infusion at the time of registration. Incentives for participation included a free designer Risen Regalia facemask, a chance to win an Amazon or Visa gift card in a raffle, or a hospital cafeteria gift card.

### 2.2 Study design

**Figure 1:**
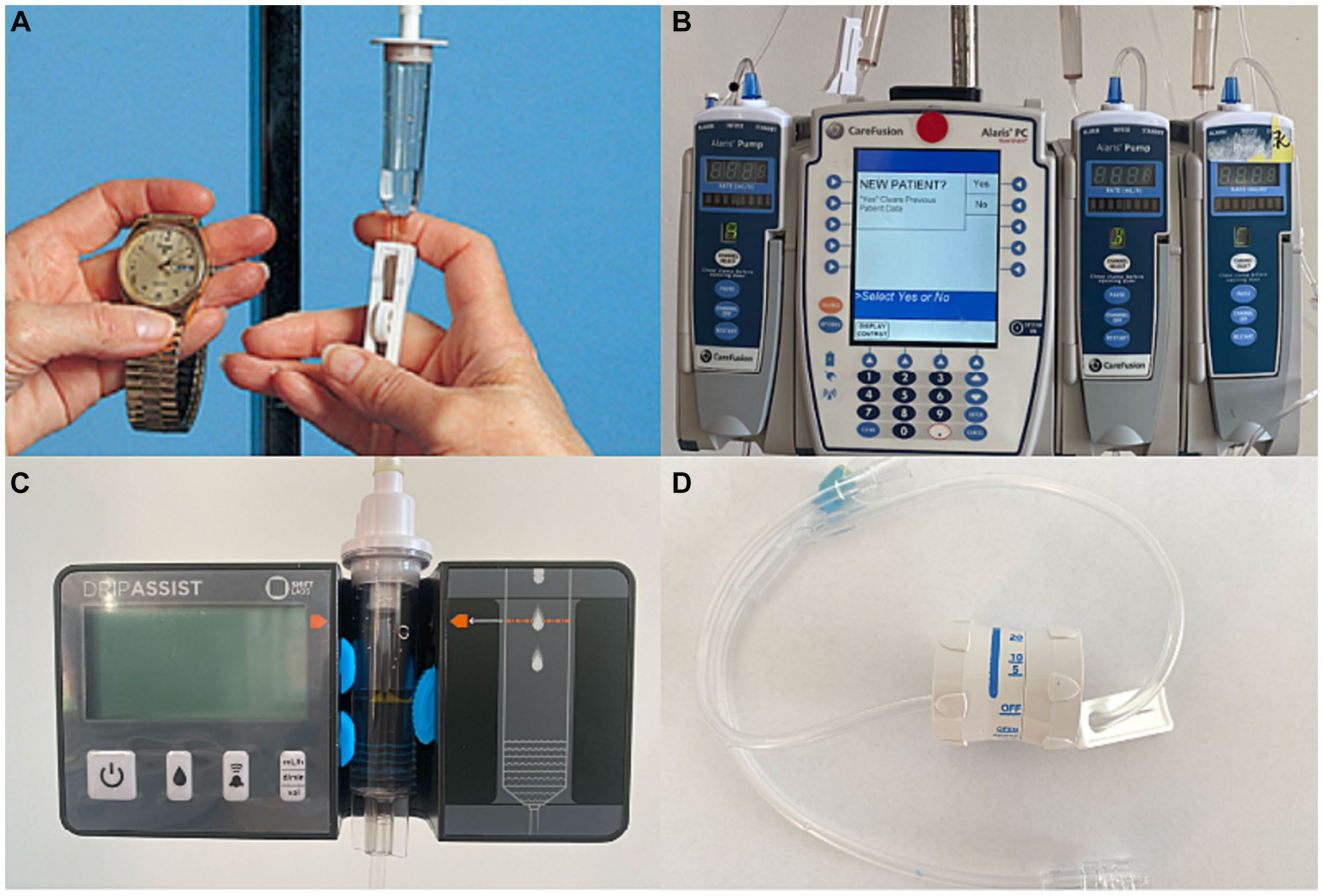
Intravenous (IV) infusion methods. **(A)** Drop counting. This method is the most traditional way of delivering infusions in low-resource settings. Gravity is used as the driving force to administer the IV fluid through tubing with a drip chamber. **(B)** IV infusion pump. The Alaris IV infusion pump is a relatively expensive device that administers fluid in controlled amounts using a built-in software interface. **(C)** The DripAssist is a small, lightweight device that operates with one AA battery. The rate of fluid infused is automatically monitored by the device itself. The DripAssist device also uses an alarm technology that alerts the user of a significant change in drip rate. It provides a display of the rate as milliliters/h, drops/min, or total milliliters. **(D)** The manual flow regulator is a simple cylindrical device that is attached to the IV tubing and allows the user to set the flow to a constant rate. This device may make the infusion process easier, although the drip rate must be monitored occasionally.

Healthcare nurses and trainee volunteers were asked to operate 5 different IV infusion methods (Figure 1). Each method was performed at one of 5 stations: manually counting drops (station 1), using an IV manual flow regulator (station 2), using an Alaris IV infusion pump: BD Alaris™ Pump Module (station 3), using the DripAssist device: DripAssist Infusion Rate Monitor from Shiftlabs (station 4), and using the DripAssist device and the IV manual flow regulator together (station 5). Each station had 1 to 3 IV bags, an IV pole, an infusion set, timers, and 3 graduated cylinders. The IV bags contained saline solution, and all the parameters, including IV fluid bag, fluid type, and fluid viscosity, were kept the same to avoid confounding bias. Study team members were aware that 1 mL in different solutions could lead to different drop count measurements; thus, IV saline was the only IV fluid used in the study.

For accurate volume measurements, the fluid was collected in a 250 mL graduated cylinder clamped to a stand.

### 2.3 Data collection

Each data collector was trained on how to operate equipment at each station and how to demonstrate each station to study participants. We employed beta-testing with 10 participants to refine our recruitment, enrollment, equipment use, and data collection methods. These participants were not reflected in the participant demographics or data analysis. Outcomes included station setup time, drip rate accuracy, drip rate precision, and cost of using these devices in an acute care setting. Participants completed a questionnaire after the trial in which they were asked to rate the operability, understandability, and perceived time consumption of each method on a 5-point Likert scale. Participants also had the option to leave qualitative comments on the survey, which we separately analyzed for common themes.

The accuracy and precision of each device was measured by comparing drip rates. The participants were asked to set each device to a specific drip rate: 240, 120, and 60 mL/h. After 10 min, the researcher measured the total volume collected in the cylinder and calculated the drip rate with the following formula:


$$\mathrm{drip}\;\mathrm{rate}=\frac{volume\;\left(\mathrm{mL}\right)}{\mathrm{time}\;\left(\min\right)}$$

Study team members were aware that 1 mL in different solutions could indicate a different drop count; thus, IV saline was the only IV fluid used in the study.

The accuracy of the setup was independently determined by 2 researchers, who measured the volume of the fluid collected in the cylinder after 10 min. The difference between expected and calculated drip rate determined the accuracy level. The range of the standard deviation determined the precision, with a wider standard deviation demonstrating lower precision and a narrower standard deviation showing higher precision.

Setup time was recorded with a stopwatch and included the time to determine the given flow rate until the time that the participant was satisfied with the setup and informed the researcher. Setup time did not include the time to set up the bag (which was measured separately). We averaged and reported the bag setup times for those who set up the bag before setting up the infusion rate.

To accurately represent cost of care in an ICU setting, 2 investigators independently collected data about cost using public health resources in Senegal and India (21–25).

### 2.4 Cost-effectiveness modeling in Senegal and India

To determine the cost-effectiveness of the infusion methods in low-resources settings, we created a model of a hypothetical ICU in India and Senegal in which the methods would be deployed. The model assumed that a patient would enter the ICU and receive intravenous sedation therapy for 3 days. The cost measures were the costs of materials for each infusion method, time/hly wages for nurses and physicians, hospital stay, days on ventilator, and costs of sedatives (midazolam and fentanyl) at a rate of 10 mL/h (Table 1). We also made the assumption that the accuracy of the infusion method would be reflected in a patient’s sedation score. A traditional Richmond-Agitation Sedation Scale (RASS) (26) ranges from +4 to −5. To make mathematical calculations and comparisons between the infusion methods possible, corresponding numerical equivalents (ranging from 1 to 10) were substituted for each RASS score (Table 2).

**Table 1:** Costs associated with ICU care in Senegal and India.

Parameter	India	Senegal
Cost of ICU ventilator/day		
Public hospital	$7.50	NA^a^
Private hospital	$39.13	NA^a^
Cost of ICU stay/day		
Public hospital	$33.33 all inclusive	$55
Private hospital	$93.75 all inclusive	$330 to $400
Typical ICU bed count		
Public hospital	5 to 6	4 to 7
Private hospital	18	4 to 7
Nurse salary		
Public hospital	$740 to $2,100 per month (basic+DA)	$15 for 12 h
Private hospital	$5.30 per hour	NA
Physician salary		
Public hospital	Level 10 to 15: $740 to Rs 4,200 per month (basic +NPA + DA + TA)	$50 for 24 h
Private hospital	$14 per hour	$62 for 24 h
Cost of sedative/pain management medications		
Fentanyl	$0.45 per 100 mcg ampule	$14.00 per box
Ketamine	$0.52 per vial	$4.60 per box
Midazolam	$0.35 per vial	$4.67 per box
Morphine	$0.37 per ampule	$152.00 per box
PCM	$0.37 for 1 g infusion	NA
Diclofenac	$0.25 for 75 mg	NA
Ketolorac	$0.37 per injection	NA
Tramadol	$0.50 per injection	NA

ICU, intensive care unit; NA, not applicable. PCM, paracetamol. Rs, Rupees. NPA, Non-practicing allowance. DA, Dearness allowance. TA, Traveling allowance.

a There is no structure in Senegal that bills for ventilator use.

**Table 2:** Richmond-Agitation Sedation Scale (RASS).

RASS	Description	Numerical equivalent
+4	Combative	1
+3	Very agitated	2
+2	Agitated	3
+1	Restless	4
0	Alert & calm	5
-1	Drowsy	6
-2	Light sedation	7
−3	Moderate sedation	8
−4	Deep sedation	9
−5	Unarousable	10

Cost-effectiveness of the infusion methods was compared by using the incremental cost-effectiveness ratio (ICER). ICER is calculated by dividing the difference in total cost (incremental cost) by the difference in the chosen measure of health outcome or effect (incremental effect) to provide a ratio of “extra cost per extra unit of health effect” for the more expensive therapy versus the alternative. Suboptimal sedation in our model would be associated with lower quality-adjusted life years (QALY) (27). Therefore, we used QALY as our health effect in the calculation of ICER.

### 2.5 Data analysis

Quantitative variables were compared by a 2-way ANOVA, testing with replication for all different devices. ANOVA testing was completed with WINKS WDA 7.0.9 (Texasoft, Dallas TX). The confidence level for hypothesis testing is 95%, and the α level is 0.05. For each dataset, the F-distribution and *p* value were determined. ANOVA was used for station setup time.

Kruskal Wallis tests were used to assess differences between types of practitioners and for setup time, flow rate accuracy, and participant ratings of each station. The Newman–Keuls multiple comparison test was used to assess statistical significance between stations for setup time and participant ratings. The statistical software used was SAS version 9.4 (SAS institute, Cary, NC).

For the qualitative, open-ended responses in the survey, data were analyzed by noting themes using content analysis as determined by 2 independent researchers.

## 3 Results

**Figure 2:**
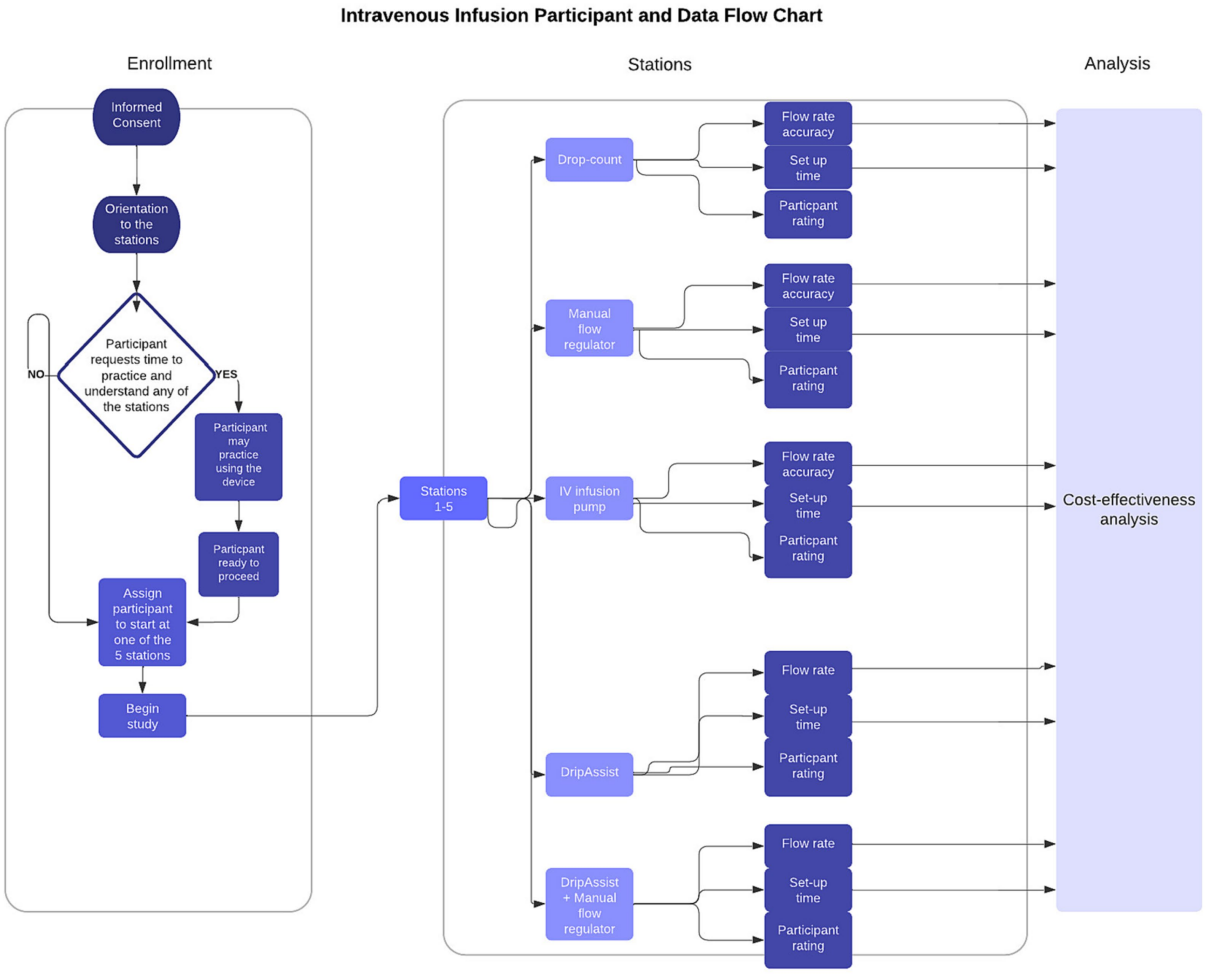
Study participant and data flow chart. Fifty-four participants (*n* = 54) enrolled and completed the study at all 5 stations and filled out a survey to rate each station. Data collected from participant flow rate accuracy and participant set-up time were used for cost-effectiveness analysis.

The study included 54 nurse, resident, and medical student volunteers (Table 3; Figure 2). Most participants were nurses, and the average number of years in practice was less than 10 but varied by vocation. The study included health professionals with diverse backgrounds, including previous experiences in low-resource settings around the world.

**Table 3:** Participant demographics.

Demographic variable	No. (%) of participants
Age range, years	
18–25	7 (12.96)
26–35	24 (44.44)
36–50	14 (25.93)
51–65	8 (14.81)
>65	1 (1.85)
Scope/Level of practice	
Nurse	34 (62.96)
Nurse anesthetist	11 (20.37)
Nurse (other)	23 (42.59)
Doctor	20 (37.04)
Medical student	11 (20.37)
Resident	9 (16.67)
Years of practice	
0	13 (24.07)
<1	4 (7.41)
1–10	20 (37.04)
11–20	9 (16.67)
>20	8 (14.81)
Gender	
Male	11 (20.4)
Female	43 (79.6)
Practical experience in a low-resource country	
Yes	6 (11.1)
No	48 (88.9)

### 3.1 Accuracy and precision of the infusion methods

A comparison of the results from all 5 stations showed no statistically significant differences in accuracy at various flow rates of 240 mL/h (*p* = 0.878), 120 mL/hour (*p* = 0.093), and 60 mL/h (*p* = 0.105; Table 4). At the highest infusion rate, the infusion pump had the greatest accuracy of flow, with a mean (SD) of 236 (21.7) mL/h, but this value was not statistically significant from that at other stations. The infusion pump and the manual flow regulator (226 [49.0] mL/h) appeared to have the highest precision.

**Table 4:** Comparison of flow rate accuracy among infusion methods.

Parameter	Drop counting	Manual flow regulator	Alaris IV infusion pump	DripAssist	DripAssist + manual flow regulator	*p* value
Flow rate goal: 240 mL/h						
Actual flow rate, mean (SD)	273.79 (155.79)	226.56 (49.04)	236.54 (21.7)	229.45 (73.22)	221.26 (62.82)	0.878
Mean deviation from the goal, %	14.08	−5.60	−1.44	−4.40	−7.81	
n	53	54	54	53	54	
Flow rate goal: 120 mL/h						
Actual flow rate, mean (SD)	124.83 (83.75)	118.86 (25.62)	117.16 (7.68)	108.25 (40.12)	109.22 (25.84)	0.093
Mean deviation from the goal, %	4.03%	−0.95%	−2.37%	−9.79%	−8.98%	
n	36	37	37	36	36	
Flow rate goal: 60 mL/h						
Actual flow rate, mean (SD)	65.38 (51.18)	55.41 (12.19)	57.78 (10.77)	61.71 (74.37)	56.82 (25.12)	0.105
Mean deviation from the goal, %	8.97%	−7.65%	−3.70%	2.85%	−5.30%	
n	34	34	36	35	34	

IV, intravenous; SD, standard deviation.

When we analyzed the results based on residents and nurses alone, we found that at 240 mL/h, the differences in stations were significant (*p* = 0.03). In that analysis, the Alaris infusion pump’s accuracy was similar to that of the DripAssist and the DripAssist combination stations, but it was significantly more accurate than drop counting and the manual flow regulator methods.

### 3.2 Setup times

Setup time differed significantly between stations (*p* < 0.001); the manual flow regulator was the least time consuming (mean [SD], 71 [56.2] seconds), and the combined DripAssist station was the most time consuming (365 [291.2] seconds; Table 5). Setup time for the manual flow regulator was not significantly different from that of the infusion pump, which had the second fastest time of 116 (77.9) seconds. The drop-counting method (225 [187.2] seconds) was the third fastest station to set up and was significantly different from the other 4 stations. The DripAssist station and the combined manual flow regulator and DripAssist station took the most time to set up (352 [240.1] seconds and 365 [291.2] seconds, respectively), but they were not significantly different from one another. Setup time for drop counting was significantly different between types of practitioners (*p* = 0.001), with nurses taking significantly less time than others.

**Table 5:** Setup times and participant ratings for each infusion method.

Parameter	Drop counting	Manual flow regulator	Alaris IV infusion pump	DripAssist	DripAssist + manual flow regulator	*p* value
Setup time, seconds, mean (SD)^**a**^	224.83 (187.17)	70.76 (56.2)	116.07 (77.89)	351.83 (240.07)	365.39 (291.24)	<0.001
Participant rating						
Understandability, mean (SD)^**b**^	4.462 (0.917)	4.792 (0.567)	4.038 (1.176)	3.811 (1.128)	3.528 (1.31)	<0.001
Operability, mean (SD)^**c**^	3.094 (1.56)	4.692 (0.729)	4.204 (1.122)	2.843 (1.189)	3 (1.188)	<0.001
Time consuming nature, mean (SD)^**d**^	3.302 (1.367)	1.226 (0.577)	1.717 (0.907)	3.698 (1.17)	3.442 (1.243)	<0.001

IV, intravenous; SD, standard deviation.

a *n* = 54 for all methods except for DripAssist, where *n* = 53.

b Rating scale was 1 to 5, with 5 being very easy to understand. *n* = 53 for all methods except for drop counting, where *n* = 52.

c Rating scale was 1 to 5, with 5 being very easy to operate. *n* = 51 for DripAssist and DripAssist + manual flow regulator; *n* = 52 for manual flow regulator; *n* = 53 for drop counting; *n* = 54 for Alaris IV infusion pump.

d Rating scale was 1 to 5, with 5 being very time consuming. *n* = 53 for all methods except for DripAssist + manual flow regulator, where *n* = 52.

The mean bag setup time was 100 s for those who set up the bag before setting up the infusion rate.

### 3.3 Participant ratings of the infusion methods

Ratings of understandability, operability, and perceived time consumption of the infusion methods differed significantly between stations (*p* < 0.001). The manual flow regulator was considered to be the most operable, most understandable, and least time consuming, and the DripAssist stations were considered the most time consuming (Table 5).

Understandability also varied among stations (*p* < 0.001). The drop-counting method and the manual flow regulator (stations 1 and 2) were rated the easiest to understand (mean [SD], 4.5 [0.92] and 4.8 [0.57], respectively) but were not statistically different from each other. The Alaris infusion pump and the DripAssist stations were the next easiest to understand (4.0 [1.2] and 3.8 [1.1], respectively) but were not statistically different from each other. The combined DripAssist and manual flow regulator station was the most difficult to understand (3.5 [1.3]) but was not statistically different from the DripAssist alone.

Analysis of operability (*p* < 0.001) showed that the manual flow regulator was the most operable device (4.7 [0.73]) by a statistically significant margin. The Alaris infusion pump was the second most easy station to operate (4.2 [1.1]), and this again was statistically different from other methods. Stations 1, 4, and 5 were the most difficult to operate (3.1 [1.6], 2.8 [1.2], and 3.0 [1.2], respectively) and were not statistically different from each other.

For the perceived time-consuming nature of the stations (*p* < 0.001), the manual flow regulator (station 2) was rated as the least time consuming (1.2 [0.58]), and this was statistically significant. The Alaris infusion pump (station 3) was perceived as the next least time-consuming device (1.7 [0.91]) and was also statistically significant. Stations 1, 4, 5 were rated as being the most time consuming, and the differences between these 3 were not statistically significant (3.3, [1.4], 3.7 [1.2], 3.4 [1.2], respectively).

### 3.4 Content analysis of participant survey comments

Several common themes emerged from participants’ responses: perceived accuracy, time-consuming nature of method, willingness to use in low-resource settings, favorites, and suggestions for implementation in low-resource settings.

#### 3.4.1 Perceived accuracy

Participants thought that the manual flow regulator was less accurate than other methods because the flow rates required estimates that were not on the device. For example, one participant mentioned that “the dial needs to be more accurate. Cannot tell if it is set up to 240. Numbers go from 200 to 250.” Perceptions were mixed regarding whether the DripAssist was more accurate than drop counting. Although one participant preferred drop counting because the DripAssist “takes so long to set,” another participant preferred the DripAssist, “especially in critical care situations in which the rate needs to be very accurate - it’s better than nothing. However if I was just infusing fluids, I might just drop count.”

#### 3.4.2 Time consuming

Participants found the 2 DripAssist stations to be the most time consuming. One participant remarked that it was “painful,” and another pointed out that it “requires no movement,” because the device is motion-sensitive during setup. Yet another participant wrote, “I wanted to like the DripAssist, and it was more convenient than counting drops, but it is also time consuming trying to get the exact number. It’s easier for a nurse to come by and do a quick check” on the flow rate “to make sure the rate has not changed due to patient movement compared to drop count.” In comparison with other methods, “the Alaris was the fastest but using the DripAssist and manual regulator together were easy to operate and not that time consuming with practice.” Although most participants thought that the manual flow regulator alone was least time consuming, participants who liked the combination station thought that more experience with the DripAssist could decrease the time for setup and allow the accuracy to approach that of the Alaris infusion pump.

#### 3.4.3 Willingness to use in low-resource settings

Many participants stated that all of the devices could be used in a low-resource setting and that they can help improve the safety of medications administered. For example:

- “If people are taught” and oriented to the new technologies, such implementation “will be a great way to learn and it will be useful in a low-resource setting.”
- These various technology methods “will help patients get more medications in a safe and timely manner.”

#### 3.4.4 Favorites

Participants had a variety of favorites among the 5 stations for different reasons. Manual drop counting was favored because it was the “most fun.” The manual flow regulator was favored because it was not as motion-sensitive as the DripAssist and was “not bad.” The DripAssist station was favored, with reservation, because participants “would have loved this in the ICU,” but “there may be a lot of [number] rounding.” The combination of the DripAssist and manual flow regulator was favored by some because it “made me feel more confident.” The combination station was the least favorite for some participants because “positioning is key for the DripAssist” and it was “overwhelming because you have to go back and forth between devices.”

#### 3.4.5 Suggestions for implementation in low-resource settings

Participants’ suggestions for the manual flow regulator and the DripAssist ranged from improving efficiencies with the flow of patient care to using one infusion method as a backup to the other. The following are the most noteworthy feedback points gathered from the participants:

- Caution is recommended with “checking and changing tubing,” as this is one of the most important steps.
- Because of its motion sensitivity, “changes in gravity (higher/lower IV bag) affect the DripAssist” accuracy measurements.
- One suggestion targeted cost savings with DripAssists. The participant recommended running the DripAssist “to spot check-- like after running for a few minutes.” This use could allow low-resource settings to purchase fewer DripAssists.
- Another participant further suggested that in the healthcare setting, the staff could “just undershoot the rate and then supplement if necessary” as another cost-saving measure.
- A concern was raised with the manual flow regulator with regard to “[changing] the device for each patient.”

### 3.5 Cost-effectiveness analysis

The results of our study indicated that all infusion methods reached levels of accuracy that were comparable to the gold standard of the IV infusion pump (*p* = 0.8). Therefore, each method can achieve an optimal sedation state to reduce in-hospital mortality and increase quality of life. Therefore, we assigned QALY = 1 for all methods. The infusion pump would be the most expensive method in both Senegal and India, and the manual flow regulator would be the least expensive (Table 6). Therefore, the incremental cost-effectiveness ratio, or ICER, helps to relate the cost to the effectiveness of the type of IV method as illustrated below. A negative ICER indicates cost savings.

**Table 6:** Total cost per method per patient.

Infusion method	Total cost ($)**	
	Senegal	India
Drop counting	411.77	110.04
Manual flow regulator	409.56	113.26
Alaris IV infusion pump	2404.92	2108.16
DripAssist	707.76	410.57
DripAssist + manual flow regulator	711.85	414.63

IV, intravenous. **Costs are based on a 3-day stay in the ICU.

*Comparison of manual flow regulator to drop counting.*

ICER = 409.56–411.77/1 QALY = −$2.21 per QALY in Senegal.

ICER = 113.26–110.04/1 QALY = $3.22 per QALY in India.

*Comparison of DripAssist to drop counting.*

ICER = 707.76–411.77/1 QALY = $295.99 per QALY in Senegal.

ICER = 410.57–110.04/1 QALY = $300.53 per QALY in India.

*Comparison of DripAssist/manual flow regulator to drop counting.*

ICER = 711.85–411.77/1 QALY = $300.08 per QALY in Senegal.

ICER = 414.63–110.04/1 QALY = $304.59 per QALY in India.

*Comparison of IV infusion pump to DripAssist.*

ICER = 707.76–2404.92/1 QALY = −1697.16 per QALY in Senegal.

ICER = 410.57–2108.16/1 QALY = −1697.59 per QALY in India.

*Comparison of IV infusion pump to DripAssist/manual flow regulator.*

ICER = 711.85–2404.92/1 QALY = −1693.07 per QALY in Senegal.

ICER = 414.63–2108.16/1 QALY = −1693.53 per QALY in India.

*Comparison of IV infusion pump to manual flow regulator.*

ICER = 409.56–2404.92/1 QALY = −1995.36 per QALY in Senegal.

ICER = 113.26–2108.16/1 QALY = −1994.90 per QALY in India.

With the exception of the IV infusion pump, all methods met acceptable cost-effectiveness thresholds for implementation in Senegal (adjusted for purchasing power parity: $73–$1,166; actual $34–$544) or India (adjusted for purchasing power parity: $416–$2,781; actual: $115–$770) (25). The manual flow regulator appeared to have the highest cost-effectiveness. The DripAssist also met acceptable levels of cost-effectiveness in both countries and theoretically yielded cost savings when compared with implementing the IV infusion pump.

## 4 Discussion

In this study, we examined new devices for delivering IV infusions, compared their accuracy and precision, and evaluated their cost-effectiveness in low-resource settings. Additionally, we assessed the perceptions of these devices among a diverse group of healthcare workers. These participants offered additional insights into the use of the technologies in low-resource settings.

Both the DripAssist and the manual flow regulator are portable, superior low-cost alternatives to drop counting. The manual flow regulator alone was best understood and easiest to operate for the participants and had a high level of precision, making this a very favorable option for a low-resource setting. Although the DripAssist/manual flow regulator combination increased accuracy compared with manual drop counting, this combination was the most difficult to use and most time consuming, making it a less favorable option for a low-resource setting. Comments from participants suggested that the DripAssist could achieve as much accuracy as the infusion pump but that it was very sensitive, position-dependent, and more time consuming than other systems tested (Table 7).

**Table 7:** Comparison of methods based on desired metrics.

Method	Accuracy	Precision	Actual setup time	Perceived setup time	Perceived understanding	Perceived operability	Cost-effectiveness
Drop counting	+	+	+++	++	+++	+++	++
Manual flow regulator	+++	++++	+++++	+++++	+++++	+++++	+++++
Alaris IV infusion pump	+++++	++++	++++	+++++	++++	+++	+
DripAssist	++++	+	+	+	++	++	+++
DripAssist/ manual flow regulator	++++	++	+	+	++	+	+++

More + marks in a category indicates more favorable metrics for that method. IV, intravenous.

An additional consideration was that setup time for drop counting differed significantly between types of practitioners (*p* < 0.001), with nurses taking significantly less time than others. The setup time factored into our cost analysis with physician and nurse wages. Furthermore, because the Alaris infusion pump is only offered in English, language may present as another barrier to implementation in non-English speaking countries, such as Senegal.

Based on these results, we believe that healthcare providers can adapt these devices to their practice environments and thereby improve the safety of rate-sensitive IV medications over the traditional method of drop counting without significant strain on electricity, time, or personnel resources.

### 4.1 Accuracy and healthcare worker perceptions

A key finding in this study is the similarity in accuracy between the different infusion methods. As mentioned previously, the DripAssist has demonstrated accuracy and can be used in low-resource settings such as in prehospital locations and military medicine (10, 11). In addition, Couperus et al. (11) assessed the perceptions of healthcare workers who compared the DripAssist to drop counting and infusion pumps and concluded that the DripAssist was easier to use than either drop counting or infusion pumps and that the DripAssist can be accurate in a low-resource setting. Participants in our study believed the DripAssist to be as accurate as the infusion pump, but they did not find it to be easier to operate than either the infusion pump or drop counting. Of note, a disadvantage of the free drop method was the decrease in the rate as the IV bag decreases in volume. Nevertheless, participants thought that with practice, DripAssists may become easier to use. Participants cited the motion sensitivity of the DripAssist as the reason for it being the most time consuming and least operable of the devices tested.

Another consideration is the possibility of low infusion rates. In one study by Vieira *et al,* low infusion rates were shown to cause a startup delay, that is, the period between the start of the infusion at a desired infusion rate and the actual delivery of the medication. Startup delay not only delays the delivery of medication to the patient, it also creates a discrepancy between the delivered volume as recorded by the pump and the volume that is actually delivered to the patient. The startup delay was smaller at higher infusion rates. Accuracy was not affected at any of the flow rates, though total volume delivered may have been affected (28).

Additionally, our study demonstrated a significant difference in flow rate accuracy (*p* = 0.03) among the more experienced healthcare workers, namely nurses and residents. Flow rate accuracy of the DripAssist was statistically similar to that of the infusion pump and statistically different from those of the manual flow regulator and drop-counting methods. Thus, when looking to apply these methods in low-resource settings, it is important to note that nurses and residents are the backbone of the workforce. Nevertheless, medical students also learned the setup and had infusion method ratings similar to those of other health professionals.

Other considerations are that in Dakar, Senegal, the manual flow regulator is commonly used, whereas in India, manual counting of drops or milliliters is most commonly used. Despite unfamiliarity with the manual flow regular and its lower accuracy, participants demonstrated higher precision with this device than with other devices. This finding implies that healthcare settings, including those in low-resource areas, may use other metrics for deciding between methods.

### 4.2 Cost

We conducted a cost-effectiveness analysis of the 5 methods for India and Senegal as representative low-resource settings in sub-Saharan Africa and in South Asia. We included health-related costs, worker-related costs, and the direct costs of each infusion method. The health measure of interest was based on QALY after sedation in the ICU. The infusion pump appeared to be least cost-effective and the manual flow regulator appeared to be most cost effective in a low-resource environment (demonstrated for both Senegal and India). With the exception of the IV infusion pump, all methods met globally acceptable cost-effectiveness thresholds (29). It is interesting to note that in Senegal, ventilator use does not factor into the pay structure. It could be that the use of ventilators lags behind owing to lack of training; thus most ventilators in the country are not being used. Another reason could be that the use of ventilators is factored into the “oxygen need” of the patients. Conversely, in India, use of ventilators is a large contributor to cost, making it very expensive to keep a patient on the machine for more than 2 days.

### 4.3 Limitations and future areas of research

This study had some limitations. For example, not every participant had to set up an IV bag from scratch, reducing the sample size for this metric. The study was not conducted in low-resource settings, but rather in high-resource institutions, and it was not conducted at the patient bedside. It is possible that results for flow rate may differ in clinical settings, especially in low-resource areas. Finally, our cost-effectiveness analysis addressed only one outcome that was based on the accuracy of sedation flow rates, which we found differs minimally between methods. Although statistical significance was not demonstrated, statistical significance may differ from clinical significance of the variation in flow rates demonstrated in this study.

Further study of the manual flow regulator may be warranted to determine how many times it can be reused, how long on average it takes to break, and how regularly it should be tested for accuracy. In addition, future studies of the experimental methods used here should be conducted as a clinical study in a low-resource clinical setting. Such a study could be conducted in Sierra Leone, as Johns Hopkins sent 30 DripAssists there in 2020 to bolster their critical care capacity. Lastly, the devices should be trialed at other flow rates and with other fluids or medications, perhaps tailored to the most frequently used rate-sensitive medications in these settings.

### 4.4 Conclusion

Both the DripAssist and manual flow regulator are portable, superior low-cost alternatives to drop counting. The combination of the 2 devices has accuracy similar to that of manual drop counting. However, this combination was the most difficult and most time-consuming method tested in the study. The manual flow regulator alone was most understood and easiest to operate by healthcare worker participants and was determined to be the most cost effective in low-resource settings. The DripAssist followed as the next most cost-effective method. These methods can be considered for implementation in acute care environments when sedation infusions are part of IV therapy. Healthcare providers can easily adapt these devices to their practice environments and improve the safety of rate-sensitive IV medications without significant strain on electricity, time, or personnel resources.

## Data availability statement

The original contributions presented in the study are included in the article/Supplementary material, further inquiries can be directed to the corresponding author.

## Ethics statement

The studies involving humans were approved by the Johns Hopkins School of Medicine Institutional Review Board and the Howard Community College Institutional Review Board. The studies were conducted in accordance with the local legislation and institutional requirements. The participants provided their written informed consent to participate in this study.

## Author contributions

OT: Conceptualization, Data curation, Formal analysis, Investigation, Project administration, Resources, Supervision, Writing – original draft, Writing – review & editing. SA: Data curation, Formal analysis, Investigation, Validation, Writing – original draft, Writing – review & editing, Project administration. IE: Investigation, Supervision, Writing – review & editing. SW: Data curation, Investigation, Methodology, Writing – review & editing. FD: Data curation, Validation, Writing – review & editing. GD: Data curation, Formal analysis, Investigation, Project administration, Writing – review & editing. OO: Data curation, Writing – review & editing, Project administration, Visualization. KS: Data curation, Investigation, Supervision, Writing – review & editing. EA: Data curation, Investigation, Supervision, Writing – review & editing. VK: Data curation, Investigation, Writing – review & editing. JS: Conceptualization, Investigation, Supervision, Writing – review & editing. YS: Data curation, Investigation, Writing – review & editing.
