# Supplementary material for: A comparative analysis of intravenous infusion methods for low-resource environments
Source: Front Med (Lausanne). 2024 Feb 20;11:1326144. doi: 10.3389/fmed.2024.1326144 (PMC10912611; doi:10.3389/fmed.2024.1326144)
Supplement: Supplementary file 2 [file Data_Sheet_1.docx]

Appendix 1


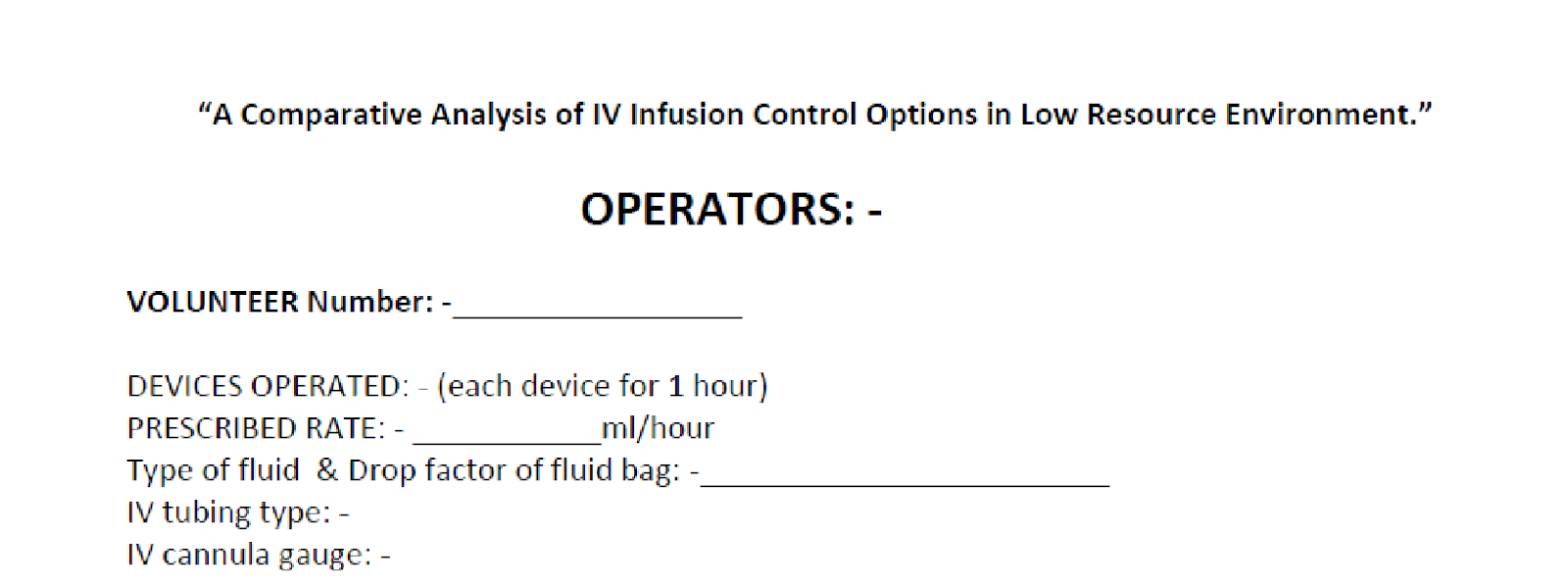


| Station | IV infusion method | Start time | End time | Completed yes/no |
| --- | --- | --- | --- | --- |
| 1 | Gravity infusion (count the drops) formula = rate (mL/hour) × drop factor (gtt/mL) |  |  |  |
| 2 | IV manual flow regulator |  |  |  |
| 3 | IV infusion pump |  |  |  |
| 4 | DripAssist |  |  |  |
| 5 | DripAssist + IV manual flow regulator |  |  |  |

Post Infusion Survey:

Please rate each station from 1 to 5 in terms of ease of understanding, ease of operation, and time consumption.

Station 1 - Drop counting– understandability

1. Very difficult to understand
2. Somewhat difficult to understand
3. Neutral
4. Somewhat easy to understand
5. Very easy to understand

Station 1 - Drop counting– ease of use (operability)

1. Very difficult to operate
2. Somewhat difficult to operate
3. Neutral
4. Somewhat easy to operate
5. Very easy to operate

Station 1 - Drop counting was:

1. Least time consuming
2. Somewhat time consuming
3. Neutral
4. More time consuming
5. Very time consuming

Station 2 - Manual flow regulator– understandability

1. Very difficult to understand
2. Somewhat difficult to understand
3. Neutral
4. Somewhat easy to understand
5. Very easy to understand

Station 2 - Manual flow regulator– ease of use (operability)

1. Very difficult to operate
2. Somewhat difficult to operate
3. Neutral
4. Somewhat easy to operate
5. Very easy to operate

Station 2 - Manual flow regulator was:

1. Least time consuming
2. Somewhat time consuming
3. Neutral
4. More time consuming
5. Very time consuming

Station 3 - Infusion pump– understandability

1. Very difficult to understand
2. Somewhat difficult to understand
3. Neutral
4. Somewhat easy to understand
5. Very easy to understand

Station 3 - Infusion pump– ease of use (operability)

1. Very difficult to operate
2. Somewhat difficult to operate
3. Neutral
4. Somewhat easy to operate
5. Very easy to operate

Station 3 - Infusion pump was:

1. Least time consuming
2. Somewhat time consuming
3. Neutral
4. More time consuming
5. Very time consuming

Station 4 - DripAssist only– understandability

1. Very difficult to understand
2. Somewhat difficult to understand
3. Neutral
4. Somewhat easy to understand
5. Very easy to understand

Station 4 - DripAssist only– ease of use (operability)

1. Very difficult to operate
2. Somewhat difficult to operate
3. Neutral
4. Somewhat easy to operate
5. Very easy to operate

Station 4 - DripAssist only was:

1. Least time consuming
2. Somewhat time consuming
3. Neutral
4. More time consuming
5. Very time consuming

Station 5 - DripAssist & manual flow regulator– understandability

1. Very difficult to understand
2. Somewhat difficult to understand
3. Neutral
4. Somewhat easy to understand
5. Very easy to understand

Station 5 - DripAssist & manual flow regulator– ease of use (operability)

1. Very difficult to operate
2. Somewhat difficult to operate
3. Neutral
4. Somewhat easy to operate
5. Very easy to operate

Station 5 - DripAssist & manual flow regulator was:

1. Least time consuming
2. Somewhat time consuming
3. Neutral
4. More time consuming
5. Very time consuming

Appendix Table 1: Excel Sheet (separate file).
